# Active head rotation in benign positional paroxysmal vertigo

**DOI:** 10.1016/S1808-8694(15)30500-0

**Published:** 2015-10-19

**Authors:** Fernando Freitas Ganança, Cristina Freitas Ganança, Heloisa Helena Caovilla, Maurìcio Malavasi Ganança, Pedro Luiz Mangabeira Albernaz

**Affiliations:** 1 ENT, PhD in Medical Sciences at UNIFESP - EPM, Adjunct Professor of Neurotology at UNIFESP - EPM. Professor and Collaborator in the Graduate Program on Vestibular Rehabilitation and Social Inclusion at UNIBAN; 2 Sciences at UNIFESP-EPM, Substitute Adjunct Professor of Hearing Disorders at the Department of Speech and Hearing Therapy at UNIFESP-EPM; 3 Associate Professor of Neurotology at UNIFESP-EPM; 4 Full Professor of Otorhinolaryngology at UNIFESP-EPM. Coordinator of the Graduate Program on Vestibular Rehabilitation and Social Inclusion at Bandeirante University in São Paulo; 5 Full Professor of Otorhinolaryngology at UNIFESP-EPM. Federal University of São Paulo - São Paulo Medical School

**Keywords:** electronystagmography, positional nystagmus, rotation, vertigo

## Abstract

Benign Positional Paroxysmal Vertigo (BPPV) is one of the most common vestibular diseases and the active head rotation test one of the most modern methods of vestibular function assessment.

**Aim:**

this study aims to verify if the active head rotation test may reveal signs of horizontal and/or vertical vestibulo-ocular reflex dysfunction in vertigo patients suspected for BPPV.

**Study design:**

retrospective series study.

**Materials and Method:**

Neurotological evaluation including computerized electronystagmography and active head rotation on the horizontal and vertical axes were conducted in 100 patients suspected for BPPV patients. Results: Isolated or associated abnormalities of the horizontal and/or vertical vestibulo-ocular reflex gain, phase and symmetry were indicative of vestibular involvement and found in 77.0% of the BPPV patients.

**Conclusion:**

the active head rotation test revealed horizontal and/or vertical vestibulo-ocular reflex dysfunctions in a relevant number of BPPV patients.

## INTRODUCTION

Positional vertigo, that is, rotational dizziness secondary to changes in the position of the head or the body, is a highly prevalent symptom observed in patients with vestibular disease. One of the labyrinthine disorders in which positional vertigo is frequently observed is benign paroxysmal positional vertigo (BPPV)^1^, ^2^, ^3^, ^4^, ^5^.

Today, the most frequently used method to detect vestibular disorders is electronystagmography (ENG). However, not all vertigo patients suspected for BPPV due to their clinical history present sings of bodily balance disorder under ENG^6^.

Passive and caloric rotational tests - conventionally used to assess vestibular-ocular reflexes (VOR) together with ENG - cannot analyze all frequency ranges applicable to vestibular function, as the frequency of the applied stimuli is too low. This is one of the reasons why abnormal results are not found for vertigo and BPPV patients in general through these tests^7^.

New methods were developed, including advanced computerized technologies, to increase the sensitiveness and accuracy of vestibular disorder clinical assessments. One of the most recent achievements is active head rotation, in which horizontal and vertical ROV are analyzed in physiological conditions, in the natural, everyday head rotation frequencies^8^.

Eye movement should be equal and opposite to head movement, in order to stabilize and maintain clarity of vision and bodily balance as subjects move out and about in their daily lives. Alterations in such pattern may be suggestive of VOR disorder^9^.

The following parameters are detected in the assessment of horizontal and vertical VOR through active head rotation: dizziness during rotation, gain (ratio between eye and head velocity), phase (delay or advancement in eye response in relation to head movement), and asymmetry (comparison between gains of head movements of opposite directions), for each stimulation frequency^10^, ^11^.

The diagnostic relevance of the active head rotation test on neurotology has been made evident both here and abroad, due to its high sensitivity in detecting functional disorders of labyrinthine bodily balance^12^, ^13^, ^14^, ^15^, ^16^, ^17^, ^18^, ^19^, ^20^, ^21^, ^22^, ^23^, ^24^, ^25^, ^26^, ^27^, ^28^, ^29^, ^30^, ^31^, ^32^.

This test does not allow the location of the lesion as peripheral or central, or to safely identify the injured side, but altered test results are indicative of vestibular system disorder^10^.

This paper aims to verify whether the active head rotation test may reveal signs of horizontal and/or vertical VOR disorder in vertigo patients suspected for BPPV.

## MATERIALS AND METHOD

This study was approved by the Research Ethics Committee of the institution in which it was performed under permit 0326/08.

This retrospective study looked at 100 consecutive vertigo patients suspected for BPPV with ages ranging 41 and 82 years, all Caucasians, 60 females and 39 males, seen during the past three years.

Suspicion of BPPV was derived from the clinical history of patients presenting recurring episodes of positional dizziness - rotational and non-rotational - whose onset was related to certain positions of the head. Episodes were of short duration, and were accompanied or not by non-neurovegetative symptoms. Other vestibular and neurologic diseases were excluded.

All patients underwent neurotological assessment, which included interview, otorhinolaryngological examination, tone threshold audiometry, voice discrimination tests, impedance tests, static and dynamic balance analysis, and computerized ENG with active head rotation.

The conduction and interpretation of audiological assessment followed the criteria defined by Mangabeira Albernaz et al.^33^. Absence of findings around hearing involvement, except when compatible with patient age and characterized as presbycusis, was a prerequisite in selecting patients with BPPV.

Computerized ENG included the following tests: eye movement calibration, positional nystagmus, spontaneous and semi-spontaneous nystagmus, saccadic movements, pendular tracking, optokinetic nystagmus, active head rotation and air caloric tests at 42 and 18 degrees centigrade, as defined by Ganança et al.^34^.

Vertigo and positional nystagmus analysis done under computerized ENG was performed using the maneuver described by Brandt^35^, also known as the Brandt, Daroff^34^, ^36^ maneuver with Frenzel goggles.

The initial position for the maneuver was the one indicated by patients as the position in which symptoms were triggered. When patients were unable to accurately identify the symptom-triggering position, the maneuver was always initiated by having the patient positioned to the right.

Active head rotation in the horizontal and vertical axes was performed with the aid of VORTEQ. VORTEQ is one of the components of a computerized ENG system (Meta 4-Channel Computerized ENG, Version 5.0, Micromedical Technologies, Inc), used in accordance with the instructions set forth on the ENG, VORTEQ and rotational chair users manual version 5.0 (1995).

Before the computerized VORTEQ ENG was performed, the patients' skin was cleaned on the sites where two horizontal channel active electrodes (one on the right outer peri-orbitary canthus and the other on the left outer peri-orbitary canthus), two vertical channel active electrodes (one above the upper eyelid on one side and the other under the lower eyelid of the same side), and one neutral electrode (placed in the frontal middle line) were placed.

A headband equipped with an angular velocity sensor was firmly strapped around the patients' heads. Patients were seated and remained with open eyes staring at a luminous stationary target in a semi-dark room, as described by Henry, Di Bartolomeo 20 and Caovilla, Ganança^13^. During the test, the electroculography amplifier recorded eye movements in the horizontal and vertical channels. The velocity sensor placed upwards in vertical position allowed the simultaneous recording of head and eye velocities in the horizontal axis. The velocity sensor placed horizontally simultaneously recorded head and eye velocities in the vertical axis. The computer program allowed the analysis o frequencies ranging between 0.5 and 8 Hz.

Patients were asked to stare at a luminous target placed one meter away from them and instructed to shake their heads to left and right at the same pace as a sound cue of variable frequency produced by an electronic metronome connected to the VORTEQ. The first set of stimuli was delivered at 1 Hz; the second from 1 to 3 Hz; and the third from 1 to 5 Hz in the horizontal axis. In the vertical axis, the sets of stimuli were delivered at 1 Hz, and 1 to 3 Hz. Each set of stimuli lasted for 15 seconds.

The sequence of three sets of stimuli at 1 Hz, 1 to 3 Hz, and 1 to 5 Hz in the horizontal axis and 1 Hz and 1 to 3 Hz in the vertical axis aimed to progressively activate the cervical muscles starting from a lower frequency and also to prevent the absence of responses to the 5 Hz stimuli, commonly seen in the 1 to 5 Hz test when only one stimulus is applied. The use of extensive stimulation also improved data consistency and allowed for artifact exclusion.

Eye movement calibration is done before horizontal and vertical axis stimulation.

The computer assessed head rotation frequency in all test cycles; cycles were grouped based on frequency ranges. All cycles close to 1 Hz (0.5 to 1.5 Hz) were grouped together; cycles close to 2 Hz (1.5 to 2.5 Hz) formed another group; and so on and so forth. Mean eye and head velocity curves were produced by the computer for each frequency group. The comparison between eye and head velocities was performed, thus producing data on gain (absolute value), phase (in degrees), and asymmetry (as a percentage) of VOR for each frequency group.

Results were presented in graphic form in the computer monitor and also in printouts. The range of variation from normal results for each VOR parameter (mean value plus or minus standard deviation) had been previously introduced by the manufacturer in the memory of the equipment. We adopted the definition of normal findings included in the equipment to characterize as normal or abnormal the three parameters of horizontal and vertical VOR under active head rotation with VORTEQ in BPPV patients. The normalcy standard on VORTEQ is similar to the definition by Caovilla, Ganança^14^, as they used the same equipment and method adopted in this study.

Isolated or associated alterations in VOR gain, phase and asymmetry were analyzed, as well as the occurrence of symptoms during the test. Abnormal findings at 1 Hz resulting from active head movement were not considered, due to the possible impact from the optokinetic and eye persecution systems.

## RESULTS

Audiological evaluation was considered normal for 81.0 % of the patients; 19.0% of the patients aged between 69 and 82 years had symmetrical bilateral sensorineural hearing loss only in higher frequencies, thus categorized as presbycusis.

When interviewed, patients complained of typical positional vertigo (96.0%) or non-rotational positional dizziness (4.0%). Positional sickness was associated with rotational and non-rotational dizziness in 38.0% of the cases. Instability not associated to positional dizziness episodes was reported by 39.0% of the patients.

Chart 1 shows vestibular function symptoms and abnormal findings in 100 BPPV cases.

**Chart 1:** Abnormal findings obtained from balance tests combined with computerized nystagmography, and active head rotation in 100 patients with benign paroxysmal positional vertigo.

Abnormal findings	Number of cases	%
Nystagmus and positional vertigo	49	49,0
Nystagmus, vertigo, and positional sickness	30	30,0
Positional vertigo	13	13,0
Vertigo or dizziness and positional sickness	8	8,0
Spontaneous nystagmus with eyes closed	1	1,0
Alterations in Saccadic Precision	4	4,0
Alterations during active head rotation	77	77,0
Alterations during air caloric test	34	34,0

All patients had one or more symptoms and/or abnormal sings when induced to experience positional nystagmus.

Combinations of vertigo and nystagmus, and vertigo, nystagmus and sickness were more prevalent than complaints of vertigo and/or sickness among patients submitted to the triggering maneuvers.

Positional nystagmus was observed in 79.0% of the BPPV patients, and was always accompanied by vertigo and/or sickness that persisted for as long as the ocular phenomenon was present. The only finding after the triggering maneuver was applied to the remaining 21.0 % was vertigo and/or sickness.

In relation to the steps of computerized ENG, we verified that spontaneous horizontal nystagmus with eyes closed and at seven degrees per second was present in 1.0% of the patients; mild saccadic movement precision alterations were observed in 4.0% of the cases; abnormal post-caloric nystagmus was found in 34.0% of the cases; and active head rotation had abnormal VOR findings in 77.0% of the patients (Chart 1). No alterations were recorded for spontaneous nystagmus with open eyes, semi-spontaneous nystagmus, pendular tracking, and optokinetic tests.

In relation to the direction of positional nystagmus cases observed through Frenzel goggles and/or recorded in ENG, when patients were submitted to the Brandt-Daroff maneuver we saw that the rotational direction occurred in 96.2% of the cases, while 41.7% of the patients also had an upward vertical component, 3.7% had a horizontal component, and 1.3% had a downward vertical component. In patients with rotational nystagmus, the counterclockwise direction was seen in 52.6% of the cases, while clockwise amounted to 47.4% of the cases.

ENG did not pick up pure rotational nystagmus. This eye movement was visualized and identified exclusively through observation of the eyes of the patients with the aid of Frenzel goggles.

The upward or downward vertical component was recorded and identified in the ENG vertical channel in all cases in which it was present.

The horizontal component of horizontal-rotational nystagmus (3.7% of the cases) was visualized with the aid of Frenzel goggles and identified in the ENG horizontal channel.

Upward oblique nystagmus to the right (1.3% of the cases) with a more intense upward vertical component was characterized after careful observation of the patients' eyes with the aid of Frenzel goggles and recorded as if it were horizontal o the right on the ENG horizontal channel and upward vertical on the ENG vertical channel.

Horizontal nystagmus to the right (1.3% of the cases) and upward vertical nystagmus (1.3%) were recognized in the ENG horizontal and vertical channels respectively with the aid of Frenzel goggles.

Positional nystagmus latency was of up to 15 seconds, duration was under 30 seconds, and fatigue due to repetition of the triggering position occurred in 98.7% of the cases. Positional nystagmus was not fatigable in 1.3% of the cases in a patient who had horizontal positional nystagmus.

Positional nystagmus velocity varied from case to case, ranging between 4 and 17 degrees per second, with a mean value of 8 degrees per second, in the lateral triggering positions.

Unilateral labyrinthine involvement was identified in 87.3% of the cases and bilateral involvement was seen in 12.7% of the patients with positional nystagmus. In bilateral cases, nystagmus was always of the pure rotational kind, counterclockwise when positioned to the right and clockwise when positioned to the left, with similar intensity.

Unilateral or bilateral involvement of the posterior semicircular canal was characterized under Brandt-Daroff maneuver in 97.5% of the cases with rotational positional, upward rotational/vertical, horizontal-rotational, and upward vertical nystagmus. In this maneuver, superior semicircular canal involvement was observed in 1.3% of the cases with downward rotational/vertical positional nystagmus and lateral semicircular canal involvement was considered in 1.3% of the cases with horizontal positional nystagmus.

In relation to abnormalities in post-caloric nystagmus in the 100 BPPV cases, vestibular hyperreflexia was found in 18% of the patients, both unilateral (8.0%) and bilateral (10.0%). Unilateral hyperreflexia occurred on the injured sides as the Brandt-Daroff maneuver was performed. Unilateral hyporeflexia was seen in 11.0% of the cases, on the same side in which there was semicircular canal involvement when the Brandt-Daroff maneuver was performed. Directional preponderance was found in 5% of the cases.

Alterations in post-caloric nystagmus were observed in cases with posterior or lateral semicircular canal involvement when the Brandt-Daroff maneuver was performed.

Mild or moderate vertigo during active head rotation was reported by 82.0% of the BPPV patients. All patients with positional nystagmus had vertigo during this test.

Abnormalities in horizontal and/or vertical VOR were observed in 77.0% of the patients with BPPV. Isolated horizontal VOR disorders were seen in 28% of the cases; vertical VOR alterations were observed in 30% of the cases; alterations in both reflexes were identified in 19% of the cases.

The prevalence of the various isolated and combined alterations in gain, phase and symmetry in horizontal and vertical VOR is described on Chart 2; concurrent parameter alterations for horizontal and vertical VOR are described on Chart 3.

**Chart2:** Number and percentage of cases with altered horizontal or vertical vestibular-ocular reflex parameters during active head rotation in 100 patients with benign paroxysmal positional vertigo.

Abnormal findings	Horizontal VOR Number of cases 4	Vertical VOR Number of cases
Increase in gain	4	5
Reduction in gain	4	3
Advancement in phase	4	3
Delay in phase	3	3
Increased gain and asymmetry	3	3
Reduced gain and asymmetry	2	2
Advanced phase and asymmetry	1	2
Phase delay and asymmetry	1	2
Increased gain and advanced phase	1	2
Increased gain and phase delay	1	1
Reduced gain and advanced phase	1	1
Reduced gain and phase delay	1	1
Increased gain, phase delay, asymmetry	1	1
Reduced gain, advanced phase, asymmetry	1	1
Total	28	30

VOR = vestibular-ocular reflex

**Chart 3:** Number and percentage of cases with altered horizontal or vertical vestibular-ocular reflex parameters during active head rotation in 100 patients with benign paroxysmal positional vertigo.

Abnormal Findings	Number of cases
Increased gain on horizontal VOR, reduced gain, and phase delay on vertical VOR	11
Reduced gain on horizontal VOR, reduced gain, and advanced phase on vertical VOR	5
Increased gain on horizontal VOR, and advanced phase on vertical VOR	3
Total	19

Alterations in gain (reduction or increase) and/or phase (advancement and/or delay) were more frequently seen than asymmetry (to the right or left, upwards or downwards) both in the horizontal and vertical VOR evaluation. Unlike gain and phase alterations, asymmetry never occurred in isolation. Asymmetry was not found either when horizontal and vertical VOR were simultaneously involved.

Patients with downward rotational/vertical or horizontal nystagmus reported vertigo during rotation and did not have altered horizontal and/or vertical VOR parameters during active head rotation.

Altered horizontal and/or vertical VOR parameters were observed in 80.5% of the 77 cases of rotational, upward rotational/vertical, horizontal-rotational, upward vertical, and oblique nystagmus that also had unilateral and bilateral involvement of the posterior semicircular canals. Altered horizontal and/or vertical VOR parameters were found in 71.4% of the 21 patients without positional nystagmus.

## DISCUSSION

According to OLeary^37^, Goebel, Rowdon^38^ and Davis^10^, decreasing pendular rotational and sinusoidal harmonic acceleration tests analyze VOR by employing only low frequency stimulate, as is also the case in caloric tests and, therefore, all such tests have limited diagnostic value as they fail to assess a wide range of frequencies inherent to vestibular function.

Gresty et al.^39^ pointed that most usual head movements occur in the frequencies between 1 and 4 Hz. According to Guitton, Volle^19^, Grossman et al.^18^ and NG et al.^27^, visual stabilization during head movement and gait is fundamentally promoted by the VOR, as the persecution and optokinetic systems are not as sensitive above 2 Hz.

Fineberg et al.^16^ stress that vertigo during head movement is very frequent. In spite of the importance of such finding, VOR is usually not analyzed with stimuli above 2 Hz, due to the technical limitations of the currently available rotational chairs.

Active head rotation uses stimuli from 0.5 to 6.5 Hz and is performed under the same physiological conditions seen in usual head movements according to Fineberg et al.^16^ and Davis^10^.

OLeary^37^ stated that day-to-day head movements induce or increase the incidence of patient-reported dizziness. Active head rotation may, therefore, be used to characterize VOR disorders better than non-physiological vestibular tests that use low-frequency stimuli.

In our study, active head rotation with VORTEQ in the horizontal and vertical axes with patients keeping their eyes open and looking at a fixed target was useful in determining other signs of vestibular disorder in BPPV patients.

Active head rotation and positional nystagmus evaluation with Frenzel goggles were the tests in which more abnormal findings were presented for BPPV patients.

The use of Frenzel goggles while assessing patients for nystagmus and other eye movements in balance tests was deemed as important by Sauron, Doubler^36^, Yokoy, Fukuda^40^, Hamid^41^ and Brandt^35^.

All 100 patients were able to go through the active head rotation test without great difficulty. Eighty-two percent of them reported mild or moderate dizziness during the test. All 79 patients with rotational nystagmus reported vertigo during rotation in this test. According to Caovilla, Ganança^12^, dizziness episodes during the test may be indicative of vestibular disorder.

Active head rotation in the horizontal and vertical axes while patients keep their eyes open looking at a fixed target revealed alterations in gain, phase and asymmetry in 77.0% of the BPPV patients, thus contributing with the diagnosis of vestibular disorder in BPPV patients. Looking only at horizontal VOR, Caovilla, Ganança^12^ noticed that 47.2% of vertigo patients with neurotological diseases of varied etiology and without altered VENG results presented a number of signs of labyrinthine involvement when performing active head rotation. These results stress the relevance of this test in confirming the existence of vestibular disorders in vertigo patients.

Corvera-Behar et al.^15^ observed increased gain and delayed phase in VOR among BPPV patients submitted to active head rotation tests with the Vestibular Autorotation Test, Western Systems Research, Inc device in the horizontal axis, and no changes on the vertical axis. They concluded that this is a valuable test in diagnosing BPPV, as ENG results are usually normal for patients suffering from this disease. As vertical VOR was normal, the authors stated that their results dispute the theory that places cupulolithiasis as the causing entity of BPPV.

In our study, alterations exclusively related to vertical VOR were found in 30.0% of the BPPV patients; alterations occurring concurrently with horizontal VOR disorder were seen in another 19.0% of the patients. Therefore, 49.0% of our BPPV patients had isolated or combined involvement of the vertical VOR, contrasting with the findings of Corvera-Behar et al.^15^.

We agree with Corvera-Behar et al.^15^ in terms of the value the active head rotation test has in confirming the presence of vestibular disorders in patients suspected for BPPV. In our study, we were also able to verify that horizontal and/or vertical VOR disorders were found in 71.4% of the 21 patients without diagnosis for positional nystagmus after ENG and careful observation with the aid of Frenzel goggles.

Considering that active head rotation with VORTEQ was only recently introduced, more studies are required to confirm our findings and to verify its comprehensive application in the realm of neurotology.

## CONCLUSION

Given the many findings observed in this study, we may conclude that active head rotation with open eyes and a fixed target could identify signs of horizontal and vertical vestibular-ocular disorder in a relevant part of the population of patients suspected for BPPV.
